# Clinical covariates of self-harm and suicidality in a community sample of Irish Travellers

**DOI:** 10.1192/j.eurpsy.2023.2363

**Published:** 2023-07-19

**Authors:** K. Tong, M. McGovern, R. McManus, J. O’Brien, A. M. Doherty

**Affiliations:** 1 National Forensic Mental Health Service, Central Mental Hospital, Dundrum; 2 Department of Psychiatry, School of Medicine & Medical Science, University College Dublin; 3 Department of Psychiatry, Mater Misericordiae University Hospital; 4 National Traveller Mental Health Service, Exchange House Ireland, Dublin, Ireland

## Abstract

**Introduction:**

Irish Travellers are an indigenous minority group in Ireland. Health inequalities have been widely reported within the Traveller community, with a shorter life expectancy of 11 years less than the general population. Travellers also have higher mortality rates of 3.5 times higher than the general population in Ireland. Suicide is a serious problem in the Traveller community with a suicide rate of 11% among Travellers: 6 times higher in women and 7 times higher in men compared with their counterparts in the general population.

**Objectives:**

There is a paucity of research into the clinical characteristics of self-harm and suicidality among Irish Travellers despite the elevated suicide rates in this community. This study aims to bridge the knowledge gap in the mental health of Irish Travellers, focusing on the clinical factors associated with self-harm and suicidality in a community sample of Irish Travellers.

**Methods:**

This is a cross-sectional study. Study participants completed self-report and interview-based validated questionnaires that screen for anxiety (General Anxiety Disorder assessment: GAD-7), depression (Patient Health Questionnaire: PHQ-9), and suicidality (Suicide Behaviours Questionnaire-Revised: SBQ-R and Adult Suicidal Ideation Questionnaire: ASIQ). Ethical approval was granted through the Clinical Research Ethics Committee, University College Dublin.

**Results:**

Despite an active recruitment campaign, participation rate from Irish Travellers in this study was low, with only five participants completing this study. Three were male. The mean age of the study participants was 39±14.7 years. All had pre-existing mental health diagnoses, most commonly anxiety disorder. All had at least one previous episode of self-harm and 80% had a positive family history of self-harming behaviour. No participants reported a history of alcohol or substance misuse. Over half of the participants reported severe anxiety and depressive symptoms with median GAD-7 score of 19 and PHQ-9 score of 21 respectively. All participants demonstrated significant risk of suicidal behaviour based on their SBQ-R and ASIQ scores.

**Conclusions:**

Despite elevated rates of suicidality and mental illness in this ethnic minority group, Irish Travellers demonstrated lower participation in mental health research, including this study. These recruitment challenges suggest that factors such as stigma, shame and lack of trust may be contributory. These factors may also act as barriers to them accessing mental healthcare when they develop mental health symptoms such as anxiety and depression, associated with increased risk of self-harm and suicidal behaviours. There is a need for better engagement strategies with Travellers to promote awareness into their needs and reduce mental health problems in this population.

**Disclosure of Interest:**

None Declared

